# 2-Amino-4-(4-methyl­phen­yl)-5-oxo-5,6,7,8-tetra­hydro-4*H*-chromene-3-carbonitrile

**DOI:** 10.1107/S1600536812029480

**Published:** 2012-07-04

**Authors:** Shaaban K. Mohamed, Mehmet Akkurt, Muhammad N. Tahir, Antar A. Abdelhamid, Mustafa R. Albayati

**Affiliations:** a Chemistry and Environmental Division, Manchester Metropolitan University, Manchester M1 5GD, England; b Department of Physics, Faculty of Sciences, Erciyes University, 38039 Kayseri, Turkey; c University of Sargodha, Department of Physics, Sargodha, Pakistan

## Abstract

The 4*H*-pyran ring of the title compound, C_17_H_16_N_2_O_2_, is nearly planar [maximum deviation = 0.077 (2) Å] and the cyclo­hexene ring adopts a flattened chair conformation [puckering parameters: *Q*_T_ = 0.435 (2) Å, θ = 122.0 (3)° and ϕ = 53.5 (3)°]. The 4*H*-pyran ring is almost perpendicular to the benzene ring [dihedral angle = 87.23 (8)°] and is almost coplanar with the mean plane of the cyclo­hexene ring [dihedral angle = 8.01 (8)°]. In the crystal, inversion-related mol­ecules are linked by pairs of inter­molecular N—H⋯N hydrogen bonds, forming inversion dimers with *R*_2_^2^(12) ring motifs. These dimers are further connected by N—H⋯O and C—H⋯N hydrogen bonds, forming a layer structure extending parallel to (0-12). Mol­ecules within the layers inter­act with each other *via* C—H⋯π inter­actions.

## Related literature

For the biological background to tetra­hydro-4-chromene and fused tetra­hydro-4-chromene compounds, see: Alvey *et al.* (2009 ▶); Symeonidis *et al.* (2009 ▶); Narender & Gupta (2009 ▶). For the synthesis of similar chromene compounds, see: Yadav *et al.* (2009 ▶); Mohamed *et al.* (2012*a* ▶,*b* ▶,*c* ▶). For puckering parameters, see: Cremer & Pople (1975 ▶). For standard bond lengths, *see*: Allen *et al.* (1987 ▶). For hydrogen-bond motifs, see: Bernstein *et al.* (1995 ▶).
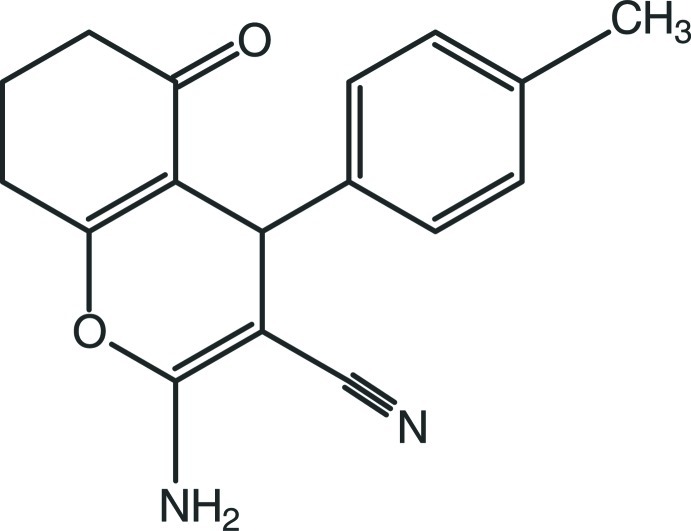


## Experimental

### 

#### Crystal data

C_17_H_16_N_2_O_2_
*M* *_r_* = 280.32
Triclinic, 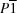

*a* = 8.5931 (9) Å
*b* = 8.7409 (14) Å
*c* = 11.0695 (19) Å
α = 72.626 (4)°
β = 70.088 (3)°
γ = 80.035 (6)°
*V* = 743.71 (19) Å^3^
*Z* = 2
Mo *K*α radiation
μ = 0.08 mm^−1^
*T* = 296 K
0.30 × 0.23 × 0.20 mm

#### Data collection

Bruker Kappa APEXII CCD diffractometer
Absorption correction: multi-scan (*SADABS*; Bruker, 2005 ▶) *T*_min_ = 0.975, *T*_max_ = 0.984
8982 measured reflections
2916 independent reflections
1704 reflections with *I* > 2σ(*I*)
*R*_int_ = 0.075

#### Refinement

*R*[*F* ^2^ > 2σ(*F* ^2^)] = 0.045
*wR*(*F* ^2^) = 0.115
*S* = 0.91
2916 reflections
191 parameters
H-atom parameters constrained
Δρ_max_ = 0.20 e Å^−3^
Δρ_min_ = −0.17 e Å^−3^

### 

Data collection: *APEX2* (Bruker, 2007 ▶); cell refinement: *SAINT* (Bruker, 2007 ▶); data reduction: *SAINT*; program(s) used to solve structure: *SHELXS97* (Sheldrick, 2008 ▶); program(s) used to refine structure: *SHELXL97* (Sheldrick, 2008 ▶); molecular graphics: *ORTEP-3 for Windows* (Farrugia, 1997 ▶) and *PLATON* (Spek, 2009 ▶); software used to prepare material for publication: *WinGX* (Farrugia, 1999 ▶) and *PLATON*.

## Supplementary Material

Crystal structure: contains datablock(s) global, I. DOI: 10.1107/S1600536812029480/sj5250sup1.cif

Structure factors: contains datablock(s) I. DOI: 10.1107/S1600536812029480/sj5250Isup2.hkl

Supplementary material file. DOI: 10.1107/S1600536812029480/sj5250Isup3.cml

Additional supplementary materials:  crystallographic information; 3D view; checkCIF report

## Figures and Tables

**Table 1:** Hydrogen-bond geometry (Å, °) *Cg*1 and *Cg*2 are the centroids of the 4H-pyran ring (O1/C8/C9/C11–C13) and the benzene ring (C1–C6), respectively.

*D*—H⋯*A*	*D*—H	H⋯*A*	*D*⋯*A*	*D*—H⋯*A*
N2—H2*A*⋯N1^i^	0.86	2.31	3.168 (2)	175
N2—H2*B*⋯O2^ii^	0.86	2.18	3.017 (2)	164
C2—H2⋯N1^iii^	0.93	2.53	3.277 (2)	138
C6—H6⋯*Cg*1	0.93	2.79	3.128 (2)	102
C7—H7*A*⋯*Cg*2^iv^	0.96	2.87	3.640 (2)	138

Symmetry codes: (i) 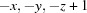; (ii) 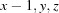; (iii) 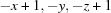; (iv) 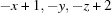.

**Fig. 1.**
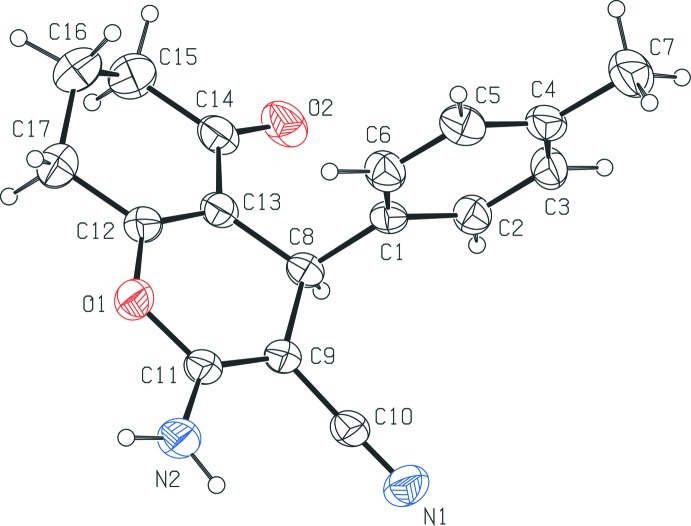
The molecular structure of the title compound showing the atom numbering scheme. Displacement ellipsoids for non-H atoms are drawn at the 30% probability level.

**Fig. 2.**
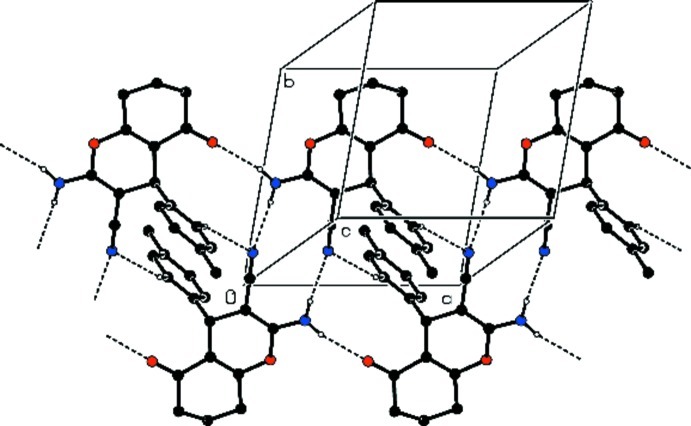
A view of the dimers formed by pairs of N—H···N hydrogen bonds, with an R22(12) motif and the N—H···O and C—H···N hydrogen bonds connecting the dimers with each other, to form a two dimensional network. H atoms not involved in hydrogen bonds have been omitted for clarity.

## supplementary crystallographic information

### Comment

Tetrahydro-4-chromene are an extensive class of bioactive compounds with antimicrobial, antifungal and antioxident properties (Symeonidis *et al.*, 2009; Narender & Gupta, 2009; Alvey, *et al.*, 2009). In a continuation to our on-going study of the synthesis and biological characterization of a new series of tetrahydro-4-chromenes (Mohamed *et al.*, 2012*a*,*b*,*c*), we report here the synthesis and crystal structure of the title compound (I).

As seen in Fig. 1, the C12–C17 cyclohexene ring of (I) is in a flattened chair conformation [puckering parameters (Cremer & Pople, 1975) are Q_T_ = 0.435 (2) Å, θ = 122.0 (3) ° and φ = 53.5 (3) °]. The O1/C8/C9/C11—C13 4*H*-pyran ring is nearly planar with a maximum deviation of 0.077 (2) Å for C8 and is almost perpendicular to the C1–C6 benzene ring [dihedral angle = 87.23 (8)°] and is almost co-planar with the mean plane of the cyclohexene ring [dihedral angle = 8.01 (8) °]. All bond lengths (Allen *et al.*, 1987) and angles of (I) are within normal ranges and are comparable to similar structures (Yadav *et al.*, 2009; Mohamed *et al.*, 2012*a*,*b*,*c*).

In the crystal, a pair of intermolecular N—H···N hydrogen bonds link the main molecules into an inversion dimer, generating an *R*_2_^2^(12) graph-set motif (Bernstein *et al.*, 1995; Table 1, Fig. 2). The dimers are further connected by N—H···O and C—H···N hydrogen bonds, forming a layer of molecules parallel to (0 - 1 2) (Table 1, Fig. 2). In addition, the layers are interconnected by weak C—H···π interactions.

### Experimental

A mixture of 168 mg (1 mmol) (4-methybenzylidene)propanedinitrile, 112 mg (1 mmol) cyclohexane-1,3-dione in presence of 61 mg ethanolamine as catalyst was refluxed in 40 ml ethanol. The reaction mixture was monitored by TLC till completion after 6 h. A solid product was deposited on cooling at room temperature and collected by filtration. The crude product was recrystallized from ethanol in excellent yield (89%). Single crystals suitable for X-ray analysis were grown upon slow evaporation of the solution of (I) in ethanol over two days [*M*.p.: 477 K].

### Refinement

H atoms were positioned geometrically and refined by using a riding model, with N—H = 0.86 Å and C—H = 0.93 Å (aromatic), 0.96 Å (methyl), 0.97 Å (methylene) and 0.98 Å (methine), with *U*_iso_(H) = 1.5*U*_eq_(O) for methyl groups and *U*_iso_(H) = 1.2*U*_eq_(C, N) for others.

### Crystal data

**Table:**

C_17_H_16_N_2_O_2_	*Z* = 2
*M**_r_* = 280.32	*F*(000) = 296
Triclinic, *P*1	*D*_x_ = 1.252 Mg m^−^^3^
Hall symbol: -P 1	Mo *K*α radiation, λ = 0.71073 Å
*a* = 8.5931 (9) Å	Cell parameters from 245 reflections
*b* = 8.7409 (14) Å	θ = 3.2–18°
*c* = 11.0695 (19) Å	µ = 0.08 mm^−^^1^
α = 72.626 (4)°	*T* = 296 K
β = 70.088 (3)°	Prism, white
γ = 80.035 (6)°	0.30 × 0.23 × 0.20 mm
*V* = 743.71 (19) Å^3^	

### Data collection

**Table:**

Bruker Kappa APEXII CCD diffractometer	2916 independent reflections
Radiation source: fine-focus sealed tube	1704 reflections with *I* > 2σ(*I*)
Graphite monochromator	*R*_int_ = 0.075
Detector resolution: 0.81 pixels mm^-1^	θ_max_ = 26.0°, θ_min_ = 2.0°
ω scans	*h* = −10→10
Absorption correction: multi-scan (*SADABS*; Bruker, 2005)	*k* = −10→10
*T*_min_ = 0.975, *T*_max_ = 0.984	*l* = −13→13
8982 measured reflections	

### Refinement

**Table:**

Refinement on *F*^2^	Primary atom site location: structure-invariant direct methods
Least-squares matrix: full	Secondary atom site location: difference Fourier map
*R*[*F*^2^ > 2σ(*F*^2^)] = 0.045	Hydrogen site location: inferred from neighbouring sites
*wR*(*F*^2^) = 0.115	H-atom parameters constrained
*S* = 0.91	*w* = 1/[σ^2^(*F*_o_^2^) + (0.0535*P*)^2^] where *P* = (*F*_o_^2^ + 2*F*_c_^2^)/3
2916 reflections	(Δ/σ)_max_ < 0.001
191 parameters	Δρ_max_ = 0.20 e Å^−^^3^
0 restraints	Δρ_min_ = −0.17 e Å^−^^3^

### Special details

**Table:**

Geometry. Bond distances, angles *etc*. have been calculated using the rounded fractional coordinates. All su's are estimated from the variances of the (full) variance-covariance matrix. The cell e.s.d.'s are taken into account in the estimation of distances, angles and torsion angles
Refinement. Refinement on *F*^2^ for ALL reflections except those flagged by the user for potential systematic errors. Weighted *R*-factors *wR* and all goodnesses of fit *S* are based on *F*^2^, conventional *R*-factors *R* are based on *F*, with *F* set to zero for negative *F*^2^. The observed criterion of *F*^2^ > σ(*F*^2^) is used only for calculating -*R*-factor-obs *etc*. and is not relevant to the choice of reflections for refinement. *R*-factors based on *F*^2^ are statistically about twice as large as those based on *F*, and *R*-factors based on ALL data will be even larger.

### Fractional atomic coordinates and isotropic or equivalent isotropic displacement parameters (Å^2^)

**Table:**

	*x*	*y*	*z*	*U*_iso_*/*U*_eq_	
O1	−0.07181 (12)	0.43638 (14)	0.69952 (11)	0.0628 (4)	
O2	0.48905 (15)	0.45532 (14)	0.66064 (13)	0.0747 (5)	
N1	0.17338 (17)	0.0029 (2)	0.50277 (16)	0.0766 (7)	
N2	−0.17035 (15)	0.27899 (18)	0.62146 (15)	0.0698 (6)	
C1	0.31349 (18)	0.13607 (18)	0.74881 (16)	0.0458 (6)	
C2	0.46472 (18)	0.04982 (19)	0.71440 (17)	0.0543 (6)	
C3	0.5138 (2)	−0.0768 (2)	0.8064 (2)	0.0625 (7)	
C4	0.4143 (2)	−0.1234 (2)	0.93584 (19)	0.0590 (7)	
C5	0.2636 (2)	−0.0370 (2)	0.97073 (18)	0.0657 (7)	
C6	0.2129 (2)	0.0905 (2)	0.87951 (18)	0.0600 (7)	
C7	0.4678 (3)	−0.2645 (2)	1.0359 (2)	0.0882 (9)	
C8	0.25982 (17)	0.27363 (18)	0.64590 (16)	0.0469 (5)	
C9	0.11699 (18)	0.23365 (18)	0.61288 (15)	0.0478 (6)	
C10	0.14572 (18)	0.1067 (2)	0.55221 (17)	0.0546 (6)	
C11	−0.03611 (19)	0.3100 (2)	0.64164 (16)	0.0510 (6)	
C12	0.0570 (2)	0.49540 (19)	0.71581 (16)	0.0532 (6)	
C13	0.21195 (19)	0.42714 (18)	0.68865 (16)	0.0501 (6)	
C14	0.3443 (2)	0.5090 (2)	0.69385 (17)	0.0606 (7)	
C15	0.2972 (3)	0.6633 (2)	0.7339 (2)	0.0896 (10)	
C16	0.1214 (3)	0.6772 (3)	0.8221 (2)	0.0916 (10)	
C17	−0.0012 (2)	0.6426 (2)	0.7642 (2)	0.0730 (8)	
H2	0.53500	0.07760	0.62750	0.0650*	
H2A	−0.16480	0.20220	0.58610	0.0840*	
H2B	−0.26260	0.33580	0.64380	0.0840*	
H3	0.61690	−0.13230	0.78020	0.0750*	
H5	0.19410	−0.06510	1.05790	0.0790*	
H6	0.11000	0.14640	0.90610	0.0720*	
H7A	0.46800	−0.22940	1.11020	0.1320*	
H7B	0.57750	−0.30710	0.99470	0.1320*	
H7C	0.39190	−0.34660	1.06630	0.1320*	
H8	0.35430	0.29220	0.56420	0.0560*	
H15A	0.31220	0.75200	0.65440	0.1080*	
H15B	0.37140	0.67300	0.78000	0.1080*	
H16A	0.09460	0.78500	0.83470	0.1100*	
H16B	0.11120	0.60230	0.90860	0.1100*	
H17A	−0.10870	0.62860	0.83160	0.0880*	
H17B	−0.01380	0.73320	0.69100	0.0880*	

### Atomic displacement parameters (Å^2^)

**Table:**

	*U*^11^	*U*^22^	*U*^33^	*U*^12^	*U*^13^	*U*^23^
O1	0.0490 (6)	0.0667 (8)	0.0832 (9)	0.0047 (6)	−0.0251 (6)	−0.0344 (7)
O2	0.0619 (8)	0.0720 (9)	0.0993 (11)	−0.0152 (7)	−0.0439 (7)	−0.0076 (8)
N1	0.0584 (9)	0.0903 (12)	0.1034 (14)	0.0059 (8)	−0.0308 (9)	−0.0570 (11)
N2	0.0474 (8)	0.0865 (11)	0.0946 (12)	0.0048 (7)	−0.0334 (8)	−0.0434 (10)
C1	0.0436 (8)	0.0481 (10)	0.0555 (11)	−0.0082 (7)	−0.0234 (8)	−0.0154 (8)
C2	0.0481 (9)	0.0560 (11)	0.0611 (12)	−0.0029 (8)	−0.0190 (8)	−0.0165 (9)
C3	0.0561 (10)	0.0559 (11)	0.0830 (15)	0.0054 (8)	−0.0338 (10)	−0.0200 (11)
C4	0.0720 (12)	0.0509 (11)	0.0668 (14)	−0.0103 (9)	−0.0387 (11)	−0.0105 (10)
C5	0.0681 (12)	0.0726 (13)	0.0562 (12)	−0.0116 (10)	−0.0232 (9)	−0.0085 (10)
C6	0.0503 (9)	0.0679 (12)	0.0623 (13)	−0.0004 (8)	−0.0202 (9)	−0.0166 (10)
C7	0.1123 (17)	0.0642 (13)	0.0970 (16)	−0.0023 (12)	−0.0601 (13)	−0.0042 (12)
C8	0.0406 (8)	0.0533 (10)	0.0510 (10)	−0.0051 (7)	−0.0192 (7)	−0.0128 (8)
C9	0.0471 (9)	0.0521 (10)	0.0512 (10)	−0.0020 (7)	−0.0218 (7)	−0.0168 (9)
C10	0.0412 (9)	0.0674 (12)	0.0648 (12)	0.0009 (8)	−0.0240 (8)	−0.0252 (10)
C11	0.0471 (9)	0.0579 (11)	0.0553 (11)	−0.0020 (8)	−0.0227 (8)	−0.0184 (9)
C12	0.0593 (10)	0.0506 (10)	0.0574 (11)	−0.0043 (8)	−0.0262 (8)	−0.0154 (9)
C13	0.0554 (10)	0.0466 (10)	0.0542 (11)	−0.0068 (8)	−0.0278 (8)	−0.0072 (8)
C14	0.0668 (12)	0.0536 (11)	0.0692 (13)	−0.0112 (9)	−0.0391 (10)	−0.0027 (9)
C15	0.1010 (16)	0.0667 (14)	0.129 (2)	−0.0138 (12)	−0.0598 (15)	−0.0332 (14)
C16	0.1157 (19)	0.0743 (15)	0.1070 (18)	−0.0041 (13)	−0.0471 (15)	−0.0426 (14)
C17	0.0797 (13)	0.0606 (12)	0.0857 (15)	0.0013 (10)	−0.0297 (11)	−0.0281 (11)

### Geometric parameters (Å, º)

**Table:**

O1—C11	1.377 (2)	C12—C13	1.334 (2)
O1—C12	1.383 (2)	C13—C14	1.470 (3)
O2—C14	1.222 (2)	C14—C15	1.492 (3)
N1—C10	1.146 (2)	C15—C16	1.501 (3)
N2—C11	1.332 (2)	C16—C17	1.516 (3)
N2—H2A	0.8600	C2—H2	0.9300
N2—H2B	0.8600	C3—H3	0.9300
C1—C8	1.518 (2)	C5—H5	0.9300
C1—C2	1.378 (2)	C6—H6	0.9300
C1—C6	1.386 (2)	C7—H7A	0.9600
C2—C3	1.378 (3)	C7—H7B	0.9600
C3—C4	1.374 (3)	C7—H7C	0.9600
C4—C7	1.515 (3)	C8—H8	0.9800
C4—C5	1.375 (3)	C15—H15A	0.9700
C5—C6	1.382 (3)	C15—H15B	0.9700
C8—C9	1.514 (2)	C16—H16A	0.9700
C8—C13	1.500 (2)	C16—H16B	0.9700
C9—C10	1.407 (2)	C17—H17A	0.9700
C9—C11	1.347 (2)	C17—H17B	0.9700
C12—C17	1.483 (2)		
			
C11—O1—C12	118.57 (13)	C15—C16—C17	112.04 (18)
H2A—N2—H2B	120.00	C12—C17—C16	110.62 (17)
C11—N2—H2A	120.00	C1—C2—H2	119.00
C11—N2—H2B	120.00	C3—C2—H2	119.00
C2—C1—C6	117.40 (15)	C2—C3—H3	119.00
C6—C1—C8	121.94 (15)	C4—C3—H3	119.00
C2—C1—C8	120.65 (15)	C4—C5—H5	119.00
C1—C2—C3	121.21 (16)	C6—C5—H5	119.00
C2—C3—C4	121.73 (17)	C1—C6—H6	120.00
C3—C4—C7	121.45 (17)	C5—C6—H6	120.00
C3—C4—C5	117.22 (17)	C4—C7—H7A	109.00
C5—C4—C7	121.33 (18)	C4—C7—H7B	110.00
C4—C5—C6	121.66 (17)	C4—C7—H7C	110.00
C1—C6—C5	120.78 (17)	H7A—C7—H7B	109.00
C9—C8—C13	109.01 (13)	H7A—C7—H7C	109.00
C1—C8—C9	111.87 (13)	H7B—C7—H7C	109.00
C1—C8—C13	112.47 (13)	C1—C8—H8	108.00
C8—C9—C10	117.75 (14)	C9—C8—H8	108.00
C8—C9—C11	123.56 (15)	C13—C8—H8	108.00
C10—C9—C11	118.67 (16)	C14—C15—H15A	109.00
N1—C10—C9	178.20 (19)	C14—C15—H15B	109.00
N2—C11—C9	127.76 (17)	C16—C15—H15A	109.00
O1—C11—N2	110.71 (15)	C16—C15—H15B	109.00
O1—C11—C9	121.53 (15)	H15A—C15—H15B	108.00
O1—C12—C17	110.90 (15)	C15—C16—H16A	109.00
O1—C12—C13	123.06 (15)	C15—C16—H16B	109.00
C13—C12—C17	126.03 (17)	C17—C16—H16A	109.00
C8—C13—C12	122.92 (16)	C17—C16—H16B	109.00
C8—C13—C14	117.92 (15)	H16A—C16—H16B	108.00
C12—C13—C14	119.08 (15)	C12—C17—H17A	110.00
C13—C14—C15	117.94 (17)	C12—C17—H17B	109.00
O2—C14—C13	120.64 (16)	C16—C17—H17A	110.00
O2—C14—C15	121.34 (18)	C16—C17—H17B	109.00
C14—C15—C16	113.52 (19)	H17A—C17—H17B	108.00
			
C11—O1—C12—C17	173.14 (14)	C1—C8—C9—C10	−64.06 (19)
C12—O1—C11—N2	−173.77 (14)	C1—C8—C13—C14	70.02 (19)
C12—O1—C11—C9	6.5 (2)	C9—C8—C13—C12	11.5 (2)
C11—O1—C12—C13	−5.9 (2)	C9—C8—C13—C14	−165.32 (14)
C8—C1—C2—C3	−179.03 (16)	C8—C9—C11—N2	−177.03 (16)
C2—C1—C6—C5	−0.2 (3)	C10—C9—C11—O1	−179.21 (15)
C8—C1—C6—C5	178.89 (16)	C10—C9—C11—N2	1.1 (3)
C6—C1—C2—C3	0.0 (3)	C8—C9—C11—O1	2.6 (2)
C2—C1—C8—C9	110.95 (17)	O1—C12—C13—C8	−4.0 (3)
C2—C1—C8—C13	−125.98 (17)	O1—C12—C13—C14	172.80 (14)
C6—C1—C8—C9	−68.1 (2)	C17—C12—C13—C8	177.11 (16)
C6—C1—C8—C13	55.0 (2)	C17—C12—C13—C14	−6.1 (3)
C1—C2—C3—C4	0.4 (3)	O1—C12—C17—C16	162.42 (15)
C2—C3—C4—C5	−0.8 (3)	C13—C12—C17—C16	−18.6 (3)
C2—C3—C4—C7	178.95 (18)	C8—C13—C14—O2	2.0 (2)
C3—C4—C5—C6	0.6 (3)	C8—C13—C14—C15	178.87 (15)
C7—C4—C5—C6	−179.08 (18)	C12—C13—C14—O2	−174.91 (16)
C4—C5—C6—C1	−0.2 (3)	C12—C13—C14—C15	1.9 (2)
C1—C8—C9—C11	114.11 (17)	O2—C14—C15—C16	−156.13 (19)
C13—C8—C9—C10	170.93 (14)	C13—C14—C15—C16	27.0 (2)
C13—C8—C9—C11	−10.9 (2)	C14—C15—C16—C17	−51.4 (2)
C1—C8—C13—C12	−113.18 (18)	C15—C16—C17—C12	46.3 (2)

### Hydrogen-bond geometry (Å, º)

Cg1 and Cg2 are the centroids of the 4H-pyran ring (O1/C8/C9/C11–C13) and the benzene ring (C1–C6), respectively.

**Table:**

*D*—H···*A*	*D*—H	H···*A*	*D*···*A*	*D*—H···*A*
N2—H2*A*···N1^i^	0.86	2.31	3.168 (2)	175
N2—H2*B*···O2^ii^	0.86	2.18	3.017 (2)	164
C2—H2···N1^iii^	0.93	2.53	3.277 (2)	138
C6—H6···*Cg*1	0.93	2.79	3.128 (2)	102
C7—H7*A*···*Cg*2^iv^	0.96	2.87	3.640 (2)	138

Symmetry codes: (i) −*x*, −*y*, −*z*+1; (ii) *x*−1, *y*, *z*; (iii) −*x*+1, −*y*, −*z*+1; (iv) −*x*+1, −*y*, −*z*+2.
